# Aneuploidy and ploidy variation conditioned by the B chromosome of maize

**DOI:** 10.1038/s41437-025-00764-y

**Published:** 2025-04-25

**Authors:** Patrice S. Albert, Hua Yang, Zhi Gao, Cassidy DeVore, James A. Birchler

**Affiliations:** Division of Biological Sciences, University of Missouri, Columbia, MO 65211 USA

**Keywords:** Cytogenetics, Genomic instability

## Abstract

The supernumerary B chromosome of maize has a drive mechanism to maintain itself in a population despite being dispensible. This involves nondisjunction of the B centromere at the second pollen mitosis that produces the two sperm followed by preferential fertilization of the egg by the B containing sperm during double fertilization. During an introgression of the supernumerary B chromosome into the inbred line B73, an unusually high frequency of trisomies for A chromosomes was observed. Due to parallels to the High Loss phenomenon in which three or more B chromosomes in a specific genetic background cause chromosomal breakage at heterochromatic knob sites during the second pollen mitosis as well as ploidy changes, this phenomenon was revisited. Examination of pollen of the High Loss line revealed a high frequency of single sperm in the presence of the B chromosomes, which was previously not realized. Crosses to tetraploid females confirmed that the single sperm were diploid and functional but also revealed the presence of diploids with their A chromosomes derived solely from the tetraploid parent indicating a “diploid induction”. Collectively, the results reveal two backgrounds in which the B drive mechanism is not confined to this chromosome causing detrimental effects by adherence of heterochromatic knobs and apparently A centromeres at the mitosis preceding sperm development. In most genetic backgrounds this process is restricted to the B chromosome but in B73 and the High Loss line, there is spillover to the normal chromosomes in distinct ways.

## Introduction

B chromosomes are supernumerary chromosomes found in thousands of plants and animals (Jones and Rees 1982; Chen et al. 2022). They typically do not provide an advantage to the host and are dispensable. The B chromosome of maize has been studied in detail for many decades (Randolph 1941; Carlson 1978; Birchler and Yang 2021). It is present in many landraces but has been purged from most breeding lines. It is found in the wild relative, teosinte, from which it has descended into maize (Longley 1927, 1938; McClintock et al. 1981). The sequence has been determined revealing a few hundred predicted genes, many of which are expressed (Blavet et al. 2021). The maize B has an impact on the expression of genes in the normal A chromosomes (Shi et al. 2022) and will increase the rate of meiotic recombination (Ayonoadu and Rees 1968; Rhoades 1968; Hanson 1969; Nel 1973; Ward 1973a; Chang and Kikudome 1974; Carlson et al. 1993).

The maize B has a drive mechanism that acts to maintain itself in a population despite its dispensability. This mechanism consists of nondisjunction at the second pollen mitosis that produces the two maize sperm such that one sperm has two B chromosomes and the other has none (Roman 1947) (Supplementary Fig. S1). The sperm with the B chromosomes shows a preference for fertilization of the egg over the central cell polar nuclei in the process of double fertilization (Roman 1948). The preferential fertilization is controlled by the female parent and occurs in most but not all lines of maize (Carlson 1969, 1999; Brennan et al. 2023). These two processes, nondisjunction of sister chromatids and preferential fertilization, comprise the drive mechanism of the maize B, which serves to place more copies of itself in the next generation than present in the parentals. However, once a certain number of B chromosomes accumulate, the two sperm are likely to both contain one or more B chromosomes and with this occurrence, preferential fertilization no longer operates (Carlson 1978). Thus, the B chromosome has evolved to perpetuate itself but also not to become overly accumulated.

In some lines of maize, the B chromosome becomes in conflict with the normal genome. In these lines, the process that mediates the nondisjunction at the second pollen mitosis spills over to cause the heterochromatic knobs in the A chromosomes to remain adhered at the second pollen mitosis (Rhoades et al. 1967; Rhoades and Dempsey 1972). The B chromosome centromere is the site of this adherence, which causes the nondisjunction (Blavet et al. 2021). However, the knobs are interstitial in A chromosome arms and therefore when they remain adhered, chromosomal breakage occurs in the respective arms. The B centromere contains a repeated sequence array that is the target of the nondisjunction (Blavet et al. 2021), and it is related to the unit repeat of the knob arrays (Alfenito and Birchler 1993), explaining how the spillover effect could occur. In most lines, however, a distinction can be made such that only the B centromere remains adhered at the second pollen mitosis.

The maize line most thoroughly investigated for this conflict is the “High Loss” line discovered and analyzed by Rhoades and Dempsey (Rhoades et al. 1967). In this line when two or more B chromosomes are present in a microspore, the breakage of knobbed chromosomes occurs at a high frequency at the second pollen mitosis. Rhoades and Dempsey also noted an increased frequency of trisomies and triploids in this material but did not study this aspect in great detail.

In the process of introgressing B chromosomes into the maize reference line of B73 (Schnable et al. 2012), an abnormal frequency of trisomies was found once the copy number of the B chromosome was increased. This finding led us to re-examine the High Loss line to characterize its effects using stocks and technologies that have emerged in the intervening 50+ years. Genetic stocks are now available that permit the detection of dominant alleles for anthocyanin pathway genes in both the embryo and endosperm of mature kernels (Birchler and Alfenito 1993) allowing events at the second pollen mitosis to be more easily studied. A fluorescent in situ hybridization (FISH) technique has been developed that allows one to determine the identity of each of the ten pairs of A chromosomes in somatic root tip spreads (Kato et al. 2004; Albert et al. 2010). In the present work, we discovered that there is a high rate of diploid sperm production in the Rhoades High Loss line when B chromosomes are present, and we have characterized the features of this phenomenon.

## Materials and methods

### Plant material

The High Loss line with and without B chromosomes was obtained from Ellen Dempsey. The *a1 A2 Bz1 Bz2 R-scm2* W22/Mo17 tester was developed in the lab. The tetraploid, also developed in the lab, is a double cross hybrid with genomes consisting of H99, Oh43, B73, and A188.

### Fluorescent in situ hybridization (FISH)

Fluorescent hybridization onto root tip metaphase chromosomes was used to determine the number of B chromosomes present in a plant, for karyotyping the High Loss lines with and without B chromosomes, and to determine the ploidy of kernels emanating from High Loss + B pollen crossed to the tetraploid.

Root tips from recently germinated seeds (or young greenhouse-grown plants) were treated with pressurized nitrous oxide gas for about 2.5 h and fixed in 90% acetic acid for 10 min as previously described (Kato, 1999), rinsed once with 70% ethanol and then stored in fresh ethanol solution at −20 °C. Enzymatic digestion, slide preparation, and hybridization protocols are detailed elsewhere (Kato et al. 2006; Albert et al. 2010; Kato et al. 2011).

The cocktail of fluorescent probes used for karyotyping the High Loss line is described in Kato et al. (2004). The simplified cocktail used most often included three probes and DAPI (4′,6-diamidino-2-phenylindole) as a counterstain. Probes used to facilitate A chromosome identification included oligonucleotides for Centromere C [5′- 6FAM - CCTAAAGTAGTGGATTGGGCATGTTCG - 3′] and a TAG microsatellite. [5′- 6FAM - (AGT)^18^ - 3′] (Integrated DNA Technologies, Coralville, IA, USA). Probes used to identify B chromosomes included either a Texas Red labeled, nick-translated B-specific repeat probe (Alfenito and Birchler 1993; GenBank AY173950.1) or a Texas Red 5′ end-labeled telomere oligonucleotide that also binds to the B-repeat [5′ - Texas Red - (TTTAGGG)^5^ - 3′] (Integrated DNA Technologies).

### Detection of embryo color in colored kernels

Unambiguous embryo color was determined by first soaking kernels in water overnight to soften them. A razor blade was then used to slice the kernels in half, revealing the color of the embryo, or the lack thereof.

### Pollen: collection, fixation, and microscopy

Fresh pollen was harvested from High Loss plants with and without B chromosomes. The number of B chromosomes in the pollen donors was determined using root meristem tissue as described in the FISH method section. Pollen was immediately immersed in 1 mL of ice-cold fixative (3 parts ethanol, one part glacial acetic acid) in a 1.5 mL microcentrifuge tube. Samples were fixed overnight at −20 °C, rinsed twice with 70% ethanol (gravity settle or brief, low-speed centrifugation between rinses), then resuspended in fresh ethanol solution and stored at −20 °C until processed for imaging. Prior to viewing pollen, most of the ethanol was removed by transferring 1–2 μL of settled pollen to 20 μL tap water in a 200 μL tube. When the rinsed pollen had settled/centrifuged, the bottom 5 μL was removed and transferred to a microscope slide. After gently rocking the slide to disperse the pollen in the drop, 25–30 μL of full-strength DAPI in Vectashield (H1200, Vector Laboratories, Newark, CA, USA) was dropped onto the pollen and the mixture covered with a 24 × 50 mm glass coverslip. Images were saved in grayscale.

### Preferential fertilization

The method to determine whether the egg cell or the central cell is preferentially fertilized by sperm containing a B chromosome is described in Brennan et al. (2023) (Supplementary Fig. S1). The determination of the presence/absence of color in the embryo involved soaking the kernels overnight in water and then slicing them in half to examine the embryo.

### Image acquisition and processing

Images were obtained using GenASIs software (Applied Spectral Imaging, Carlsbad, CA, USA) on an Olympus BX61 fitted for fluorescence microscopy. Adobe Photoshop 2024 (Adobe, Inc, San Jose, CA, USA) functions Brightness-Contrast and/or Curves were used to adjust background relative to signal strength.

## Results

**Fig. 1:**
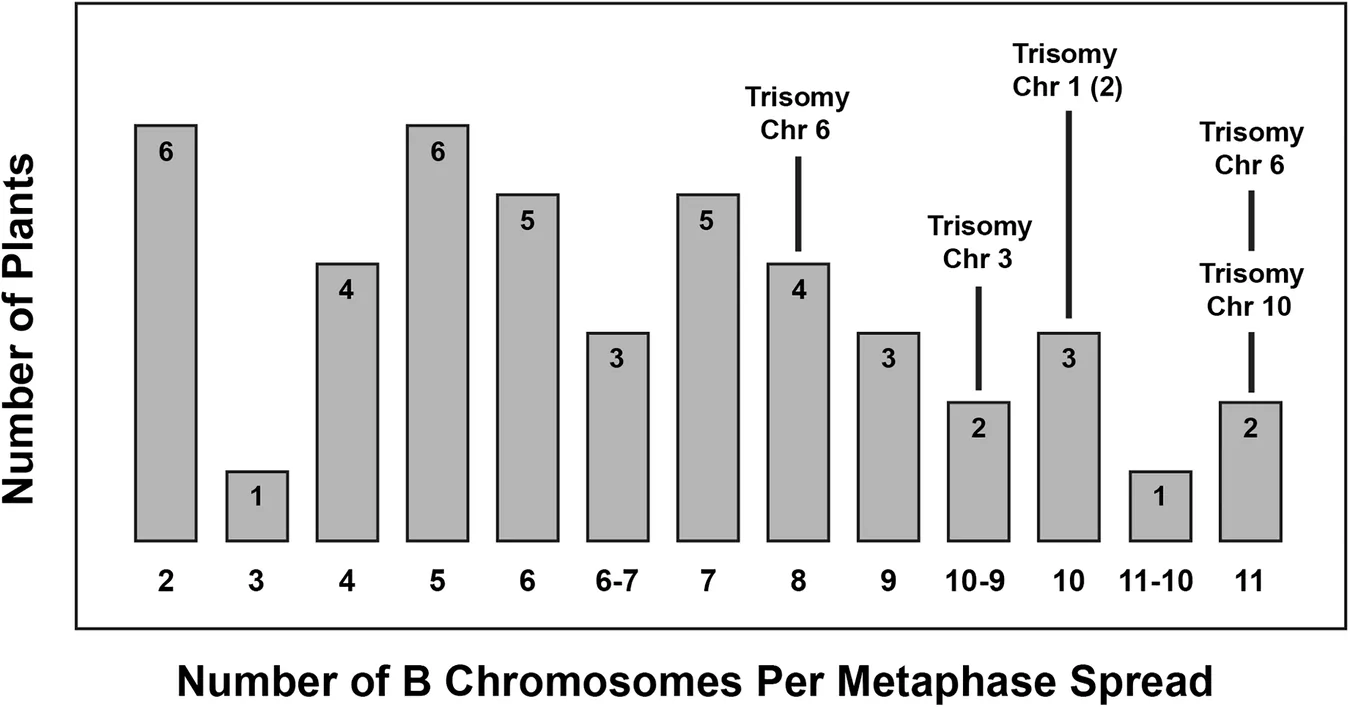
B-chromosome-induced chromosomal aberrations in maize inbred line B73. Trisomies were observed in about 13% (6/45) of the plants screened. All occurred in plants containing eight or more B chromosomes. Two of the three 10 B plants were trisomic for chromosome 1. Data are from F1’s produced by self-pollination of a 6 B plant.

In tracking the B chromosome number during an introgression into the B73 reference inbred line, a high frequency of trisomic individuals were observed. In 45 progeny screened, six individuals were found to have trisomies. These involved different chromosomes [Chromosomes 1 (x2), 3, 6 (x2), and 10] and so were not specific to any one chromosome (Fig. 1). Introgression of the B chromosome into the W22 inbred line background did not show this effect (Shi et al. 2022). This phenomenon with B73 was reminiscent of the High Loss phenomenon described by Rhoades and Dempsey (Rhoades et al. 1967; Rhoades and Dempsery 1972). This discovery prompted us to revisit the High Loss phenomenon in which B chromosomes would cause chromosomal breakage at heterochromatic knob sites in the presence of increased B chromosome numbers in that specific genetic background. A lesser studied aspect by Rhoades and Dempsey was that trisomies and triploids were also observed under these circumstances, a fact that suggested parallels with the B73 results. Having received these lines from Rhoades and Dempsey some years ago, we initiated a re-examination given that many tools are now available that could provide new insight into the effect of B chromosomes on the normal complement of A chromosomes especially with regard to the production of triploidy.

**Fig. 2:**
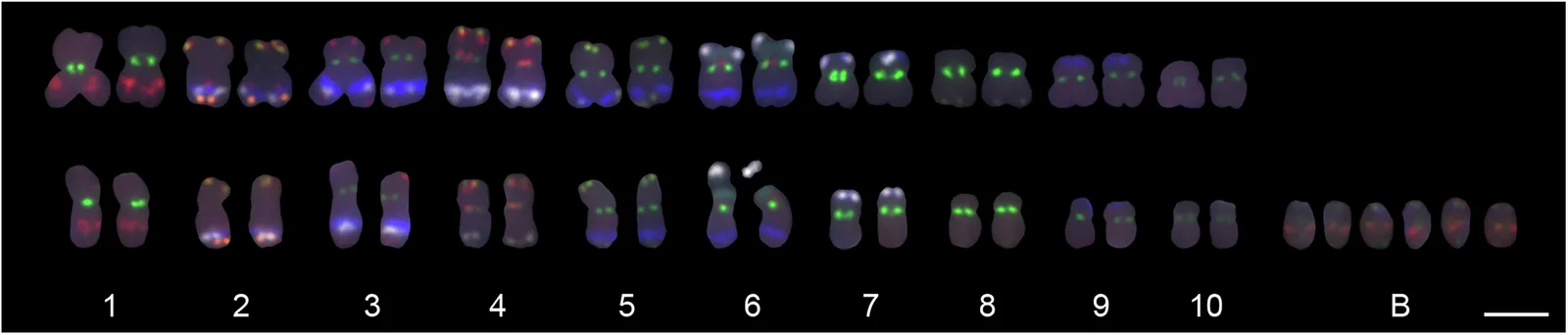
Karyotypes of High Loss without B chromosomes (top) and with B chromosomes (bottom). Characteristic of both is a large, internal heterochromatic knob on the long arm of chromosome 3. It is comprised of a 180-bp repeat (blue) and a TR-1 repeat (white). In addition to the knob probes, shown in red are the TAG microsatellite repeat and subtelomere 1.1; in teal, the 45S nucleolar organizing region; in green, centromere C and subtelomere 4-12-1; and in orange, the 5S ribosomal repeat and centromere 4. Fluorescent probes used for chromosome identification are further described in Kato et al. (2004). Scale bar, 5 μm.

**Fig. 3:**
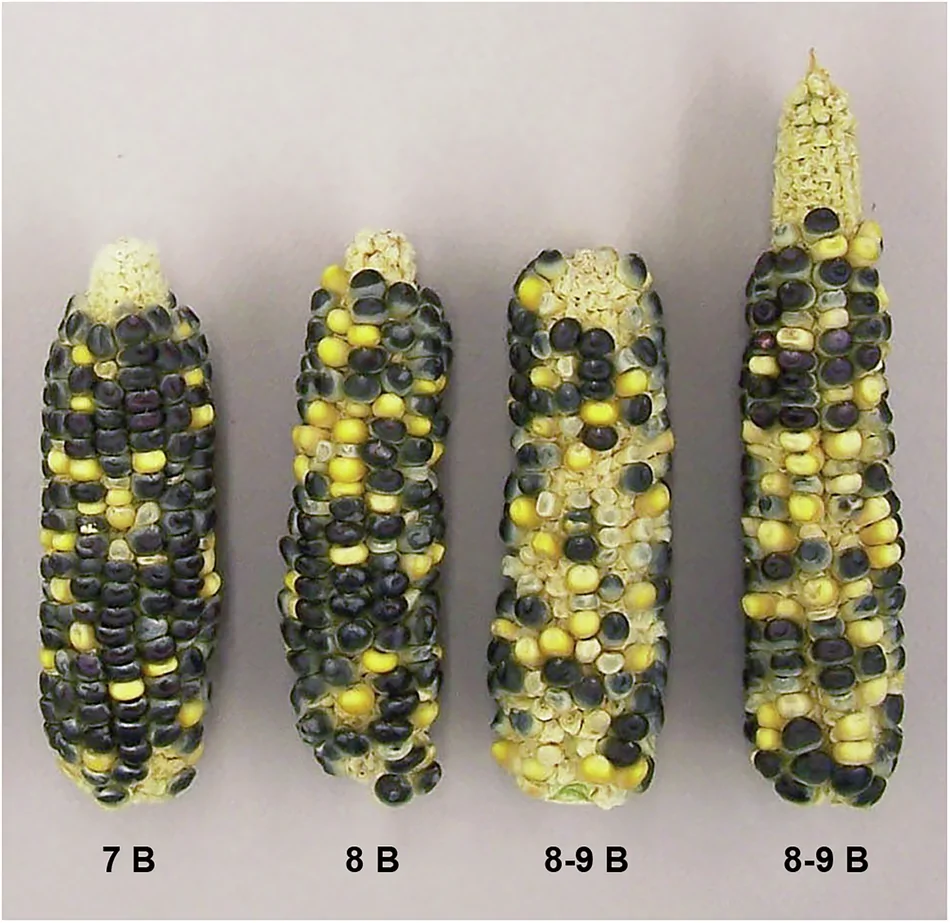
Ears from High Loss pollen with B chromosomes crossed to the 3 L *a1* tester. The High Loss line, homozygous for a dominant allele of an anthocyanin gene (*A1*), produces colored kernels. In the presence of high copy numbers of the B chromosome, chromosomal breaks that uncovered the recessive *a1* allele (*anthocyaninless*) resulted in colorless kernels (underlying yellow endosperm is visible). Numerous defective kernels were present on the ears in the higher-B-number crosses. The number of B chromosomes in the pollen donor is indicated for each ear.

In the work of Rhoades and Dempsey, there was a focus on a large knob in the long arm of chromosome 3 (3 L) to examine the parameters of the High Loss phenomenon involving breakage at knob sites in this background. FISH analysis of the two High Loss lines, with and without B chromosomes, is presented in Fig. 2. The large knob in 3 L has two types of knob repeats present. One is the canonical 180 knob repeat (Peacock et al. 1981) and the other is the TR1 repeat (Ananiev et al. 1998a). The two lines were crossed as a male parent to females that are homozygous for the recessive *anthocyaninless* (*a1*) gene that blocks the production of anthocyanin and homozygous for the dominant *R-scm2* allele of the *r1* locus that conditions pigment in both the embryo and the endosperm. This stock was produced by crossing together a W22 version and a Mo17 version so that despite the homozygous nature of *a1* and *R-scm2*, the background is hybrid producing vigorous ears. The cross of the High Loss line without B chromosomes produced F1 ears that did not uncover the recessive *a1* allele in the endosperm. In contrast, the crosses with the High Loss line with B chromosomes uncovered many kernels displaying the *a1* phenotype in the endosperm but had color in the embryos (Fig. 3) indicative of breakage of 3 L as documented by Rhoades and Dempsey. Many defective kernels were also present in these ears.

To determine if the frequency of fertilization of the embryo by the broken 3 L occurred at an equivalent frequency as occurs with the endosperm, a point noted but unresolved by Rhoades and Dempsey, the kernels from three ears were removed. The frequency of colorless (no anthocyanin) endosperm with a colored (with anthocyanin) embryo class was documented. Then the rest of the kernels, which all had colored endosperms, were imbibed in water to soften the embryos and a razor blade sliced them open to reveal whether the embryos were colored or colorless. A large fraction of these kernels had colored embryos (as well as colored endosperms) and represent cases in which the pollen grain carrying the two sperm had no 3 L chromosomal breakage. Those embryos without color represent the reciprocal fertilization in which the broken 3 L containing sperm fertilized the egg while the sperm with the intact 3 L fertilized the central cell as the progenitor to the endosperm. As noted in Table 1, the frequency of uncovering the *a1* allele in the embryo is considerably less than the frequency of uncovering the *a1* allele in the endosperm, suggesting that the sperm with the intact 3 L knob preferentially fertilizes the egg or that there is considerable attrition of the 3 L deficient embryos. A Chi^2^ test of the null hypothesis of random distribution was rejected (Chi^2^ = 87, d.f. = 1, *p* < 0.0001).

**Table 1:** Frequency of 3 L breakage in the endosperm and embryo.

Kernel phenotypes		Number of events
Colorless endosperm	Colored embryo	186
Colored endosperm	Colored embryo	234
Colored endosperm	Colorless embryo	99
Colored endosperm	Germless	62

In an experiment to corroborate this finding, the kernels from an ear of this cross involving 8 B chromosomes were removed and those with a colorless endosperm (58) versus colored endosperms (135) were separately planted in the field season of 2022. At flowering, self-pollination was performed for all plants for which that was possible. In the colorless endosperm class, self pollination of 34 plants was successful. There were also two depauperate plants that shed no pollen. Thirty segregated *A1*/*a1* indicative of the kernels being heterozygous for the two alleles. Four plants produced no kernels.

Among the colored endosperm class, due to poor survival, it was possible to self-pollinate only 33 plants with seven other plants yielding no pollen. Twenty-seven segregated *A1*/*a1* indicative of being heterozygous. Four plants were fully *a1* and semi-sterile. This is the result expected if a deletion chromosome involving 3 L was present in the sperm that fertilized the egg. Root tip chromosome examination of kernels from these ears showed no evidence of the 3 L knob, but B chromosomes were present.

Two other ears were fully colored and with variable sized kernels. This phenotype is consistent with the self-pollination of a triploid of genotype *a1*/*A1*/*A1*. Of the two viable progeny from one ear, they had 23 and 26 A chromosomes and from the second ear, the three viable seedlings had 24, 25, and 28 A chromosomes. These results are also consistent with the selfed plants being triploid as shown previously from analysis of triploids (Punyasingh 1947).

### Examination of pollen in High Loss with and without B’s

**Fig. 4:**
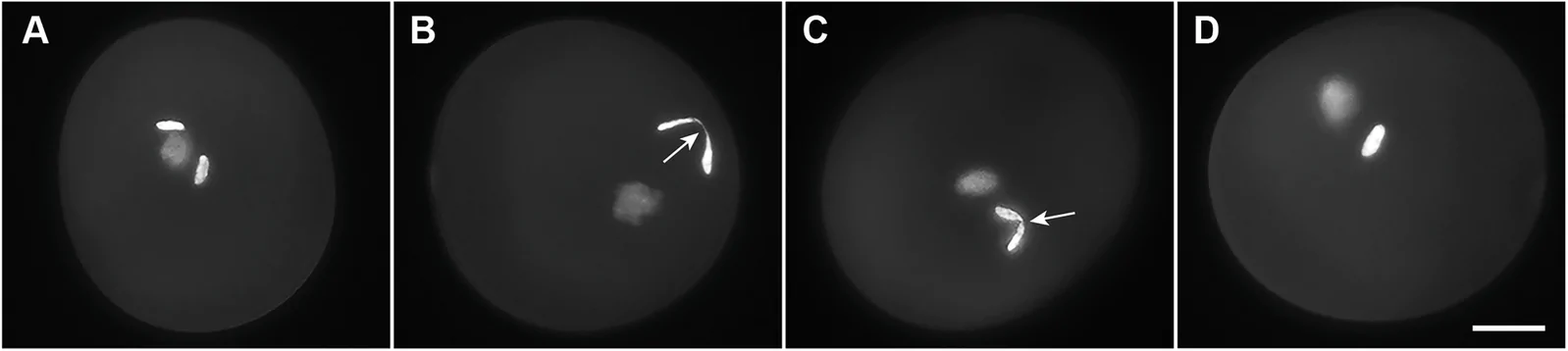
Abnormal sperm in High Loss with high numbers of B chromosomes. **A** Pollen grain with the normal complement of two haploid sperm. All examined pollen from High Loss without B’s and most pollen from High Loss with B plants displayed this phenotype. **B**, **C** Pollen grains from High Loss with B plants showing chromatin bridges (arrows) between the sperm, indicative of incomplete separation. **D** Pollen grain from a High Loss plant with B chromosomes with only one sperm, subsequently shown to be a functional diploid. In all images, the less bright object is the vegetative nucleus. Scale bar, 25 μm.

If the High Loss background causes knobs to remain adhered at the second pollen mitosis, chromatin bridges might be expected between the two sperm in pollen. To examine this question, DAPI-stained pollen was examined from the High Loss line with and without B chromosomes. Without B chromosomes, two sperm were observed in all pollen grains analyzed (*n* = 473). In the line with B chromosomes, chromatin bridges were indeed observed (Fig. 4). Interestingly, a high frequency of pollen grains was found that contained only a single sperm (Fig. 4). Table 2 shows the frequency of chromatin bridges and single sperm in material with varying numbers of B chromosomes, with higher numbers of single sperm correlated with higher B copy number.

**Table 2:** Frequency of sperm types in High Loss with and without B chromosomes.

		Sperm				
Plant	B #	Haploid	Diploid	Bridge	*n*	Diploid %
1	0	154	0	0	154	0
2	0	157	0	0	157	0
3	0	162	0	0	162	0
4	6	160	3	1	164	1.8
5	7–8	159	11	0	170	6.5
6	11–12	127	24	3	154	15.6
7	12	123	33	3	159	20.8

**Fig. 5:**
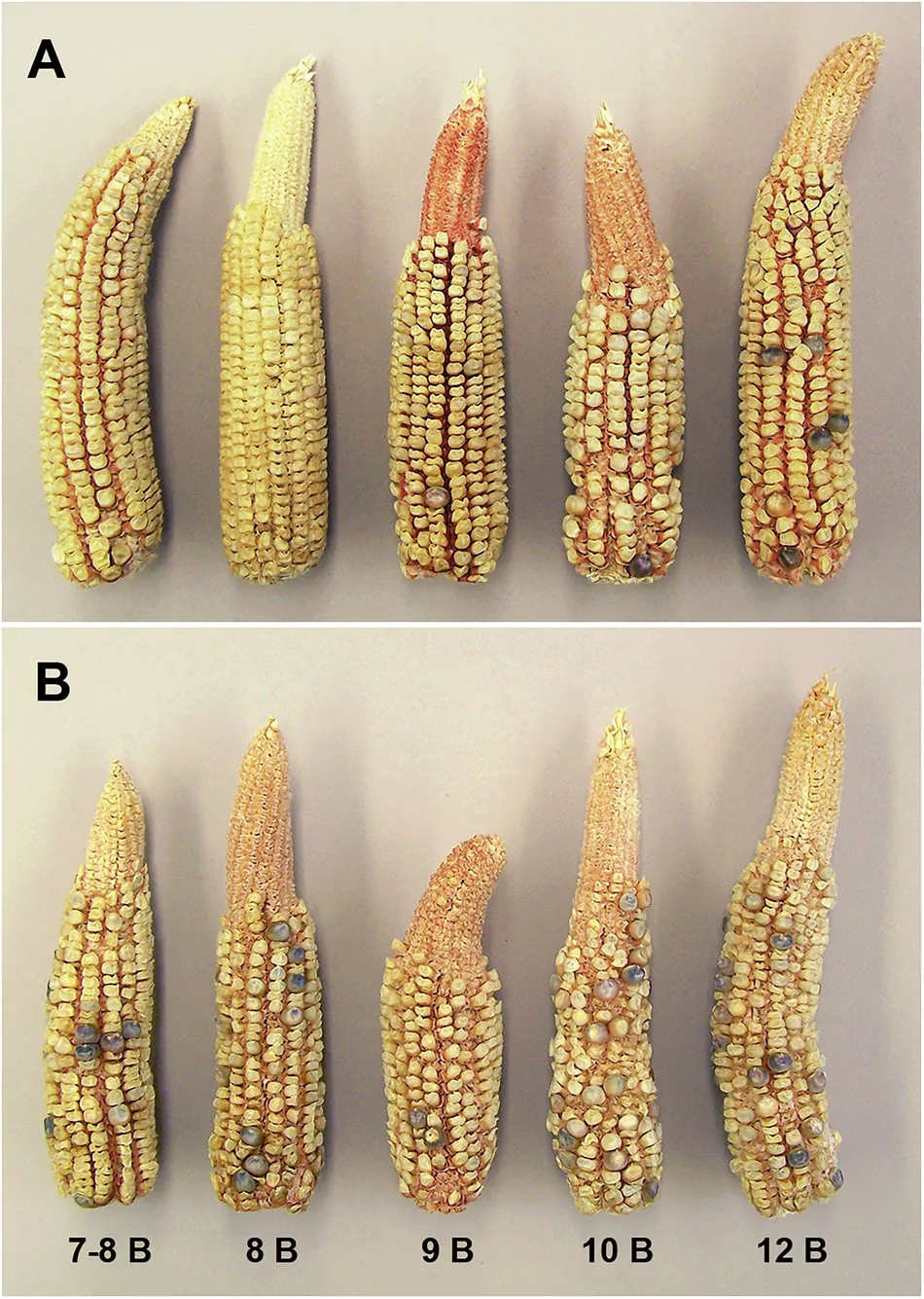
High Loss B-chromosome-induced diploid pollen is functional. **A** Ears from High Loss without B’s crossed to a hybrid tetraploid. With fertilization, the development of the resultant 5n endosperm was aborted. **B** Many fully developed kernels were produced when the High Loss pollen contained B chromosomes. A diploid sperm must have fertilized the 4n central cell to produce the viable 6n endosperm.

**Fig. 6:**
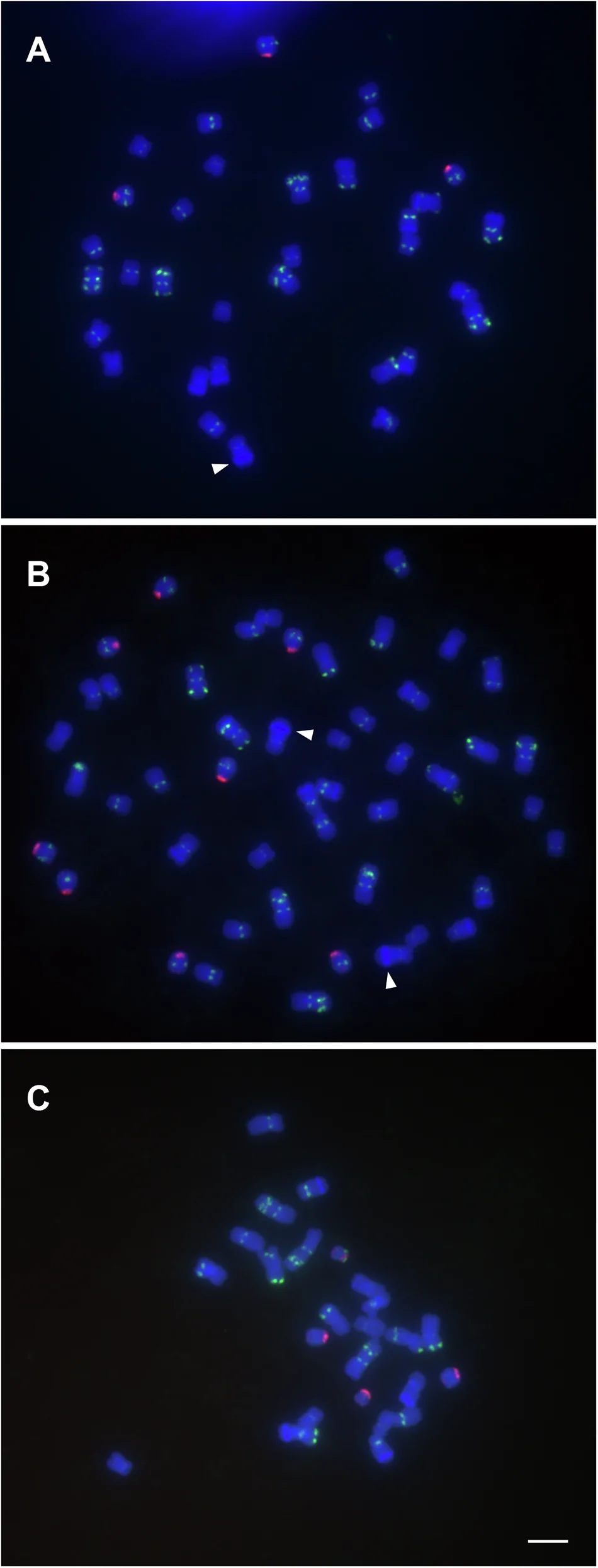
Embryo ploidy of kernels produced by crossing pollen from High Loss with B chromosomes to the tetraploid. A simplified cocktail of green fluorescent probes was used to label the A chromosomes; the B-chromosome-specific probe is shown in red. **A** Triploid (30 A chromosomes), the expected ploidy from pollination of the egg cell by a haploid sperm (one copy of High Loss chromosome 3, arrowhead). **B** Tetraploid (40 A chromosomes), the result of fertilization by a diploid sperm (two copies of High Loss chromosome 3, arrowheads). **C** Diploid (20 A chromosomes), an unexpected result. Paternal chromosomes were lost post-fertilization as evidenced by the absence of High Loss chromosome 3 and the presence of B chromosomes. Scale bar, 5 μm.

The presence of a single sperm suggested that they were diploid and could explain the recovery of triploids in the High Loss line with B chromosomes. To test whether they were indeed diploid and functional, crosses were made between a hybrid tetraploid line and High Loss with and without B chromosomes. Such interploidy crosses usually result in endosperm abortion (Randolph 1935; Cooper 1951; Birchler 1993, 2014) with only occasional kernels that are normal, which result from aberrant pollination events (Sarkar and Coe 1971; Grossniklaus 2017). Crosses with the High Loss line without B chromosomes produced mostly aborted kernels but with a few kernels with normal endosperm. Root tip analyses of these normal kernels revealed that the embryos in all cases were triploid (*n* = 16). In the crosses of tetraploid females by the High Loss line with B chromosomes, there was an approximately six-fold increase in normal endosperms (Table 3; Fig. 5). Root tip metaphase chromosome analysis of 25 of these kernels revealed tetraploid embryos (48%), triploid embryos (24%), and even diploid embryos (28%) (Table 3; Fig. 6). The tetraploid embryos contain the 3 L knob in duplicate and B chromosomes illustrating that diploid sperm are produced under these circumstances and that they can function. The triploids contained the 3 L knob and B chromosomes, suggesting that a diploid sperm fertilized the central cell to produce a viable endosperm and the accompanying egg was fertilized by a normal haploid sperm from another pollen grain in heterofertilizations. Interestingly, the diploids showed the tetraploid maternal complement without the 3 L knob, but some of them did possess B chromosomes suggesting that fertilization did in fact occur in these cases, but the paternal normal chromosomes were lost in a type of “diploid induction”. In some respects, this is analogous to haploid inducers in which the chromosomes from one parent are eliminated leaving the haploid complement from the other parent (Coe 1959; Laurie and Bennett 1988; Ravi and Chan 2010). B chromosomes introgressed into haploid inducers crossed to diploids can be transmitted to resulting haploids (Zhao et al. 2013), but whether there is an analogous process involved will require further study. If haploids are produced in crosses of High Loss to diploid females, they are unlikely to be recovered in mature kernels because the fertilization of the central cell by the single diploid sperm would result in kernel abortion and thus eliminating any cases in which the egg would develop into a haploid with or without fertilization.

**Table 3:** Embryo ploidy of kernels from tetraploid females crossed by High Loss with B chromosomes.

	Embryo Ploidy^a^			
Cross number	2N	3N	4N	NG^b^
1	0	2	3	0
2	0	1	2	2
3	2	1	0	2
4	2	1	2	0
5	1	0	3	1
6	2	1	2	0

^a^Determined by fluoresence in situ hybridization.

^b^Abbreviation for “no germination”.

Given that a single diploid sperm can only fertilize either the egg or the central cell in the process of fertilization, their recovery in triploids in diploid crosses or tetraploids in tetraploid crosses, suggests that another pollen grain is involved in the fertilization of the remaining egg or central cell. In diploid crosses, if a diploid sperm fertilizes the central cell, the endosperm will be defective and not develop. As illustrated in Fig. 3, there are many defective kernels on the ears pollinated by High Loss with B chromosomes. Only in the case that a haploid sperm fertilizes the central cell, and a diploid sperm fertilizes the egg will a triploid be recovered. In crosses to the tetraploids, only when a diploid sperm fertilizes the central cell will a normal endosperm develop and in that case the embryo can be triploid or tetraploid depending on which type of sperm fertilizes the egg. The diploid embryos apparently arise from an unknown, unusual event that eliminates the paternal gamete, but development of the maternal complement is triggered. Parthenogenetic development of diploids in tetraploid maize has previously been reported (Randolph and Fischer 1939), but the mechansism of production of those reported here is likely distinct.

### Preferential fertilization by the High Loss line

Given the extensive detrimental effects of B chromosomes in the High Loss line and the longstanding observation that some lines of maize lack preferential fertilization (Carlson 1999; Brennan et al. 2023) and that there is, in general, in landraces a negative correlation between high knob number and B chromosomes (Longley 1938), we tested whether this line might be one that has a reversed preferential fertilization to eliminate the B chromosome from the lineage given its detrimental effects. The relationship of the High Loss line to other lines of maize is unknown and indeed there is no compelling reason that reversed preferential fertilization and the detrimental effects of B chromosomes would be present in a particular line unless there was a direct connection between the two processes, but such a connection can be tested.

The High Loss line has anthocyanin pigment in the aleurone layer of the endosperm, but this background does not support anthocyanin production in the embryo of the kernel. This would ordinarily complicate determinations of preferential fertilization but a modified B chromosome carrying a transgene with a ubiquitin promoted *B-peru* transcription factor gene (Krishnaswamy et al. 2023) can be used because it conditions pigment in most plant tissues including the embryo of the kernel. Thus, the High Loss line without B chromosomes was crossed by males carrying two copies of the B-Peru B chromosome. This line will produce nearly 100% pollen with a B chromosome and the nondisjunction rate is 99% (Brennan et al. 2023). The progeny kernels were then soaked in water overnight and then sliced open to determine the frequency of the embryo color. Color would signify fertilization by the B containing sperm and absence of color would indicate that the sperm with the B chromosomes joined with the central cell. From the kernels from twelve ears totaling 1179 kernels, 789 showed color in the embryo and 390 showed no anthocyanin in the embryo. From this test, the rate of preferential fertilization is 67%. This value is within the typical range (Roman 1948; Ward 1973b; Brennan et al. 2023), indicating that the High Loss line has the standard preferential fertilization of the egg. A Chi^2^ test of the null hypothesis of random fertilization was rejected (Chi^2^ = 77.3, d.f. = 1, *p* < 0.0001).

## Discussion

The B chromosome of maize is dispensible but it has evolved a drive mechanism to be maintained in a population. This mechanism consists of nondisjunction of the B centromere at the second pollen mitosis that produces the two sperm followed by the sperm with the B chromosomes preferentially fertilizing the egg versus the central cell in the process of double fertilization. The nondisjunction occurs because a specific repeat sequence is present throughout the B centromere for which there is evidence that it is responsible for the adherence of the B centromeres at the second pollen mitosis (Blavet et al. 2021).

The repeats in and around the B centromere share a portion of homology with the knob repetitive sequence (Alfenito and Birchler 1993). In normal lines of maize, there is a distinction between the nondisjunction of the B chromosome conditioned by its centromere remaining adhered at the second pollen mitosis and the separation of knob sequences on the A chromosomes, but in the High Loss line, this distinction is removed. Because the region of the B chromosome that remains adhered is the centromere, there is no damage to the chromosome during anaphase. However, because the knobs are internal to A chromosome arms, their adherence at this mitosis will create a bridge between the poles eventually resulting in chromosome rupture. By examination of mature pollen of the High Loss line with and without B chromosomes, we were able to document examples of chromatin bridges being present between the two sperm, presumably from those cases in which the rupture was not completed.

After chromosomal breakage involving chromosome arm 3 L at the second pollen mitosis, the broken chromosome can be delivered to either the egg or the central cell and a normal chromosome will join with the other cell. Determination of embryo color in the colored endosperm class and progeny testing resolved a previously undetermined aspect in that the frequency of broken chromosomes in the embryos or endosperms is not equal. Whether this discrepancy is due to a preferential fertilization of the egg by the sperm with the normal chromosome with the 3 L knob or whether embryos with deletions are subject to attrition is not known, especially given the high frequency of defective kernels in these crosses.

In the original description of the High Loss line, the presence of a higher than normal rate of trisomies and triploids was noted (Rhoades et al. 1967; Rhoades and Dempsey 1972). Rhoades and Dempsey were not convinced that this aspect of the High Loss line was related to the chromosomal breakage aspect. Here we discovered that in the presence of B chromosomes, there is a high rate of single sperm. In those cases, all chromosomes remain together at the second pollen mitosis. Crosses to tetraploids established that these sperm were diploid and could function, and that the accompanying fertilization comes from a different pollen grain.

Previous work has shown that this can occur. In experimental production of diploid sperm to produce triploid plants by treatment of immature tassels with trifluralin that blocks mitosis, pollinations were made on subsequent days with differently marked lines (Kato 2001; Auger et al. 2005). In some cases, the egg and its accompanying central cell in a female gametophyte were fertilized on different days by different sperm from different pollen grains with different genetic markers in a process similar to heterofertilization (Kato 2001; Auger et al. 2005). When the same procedure using pollen from treated diploid plants was placed on the silks of tetraploids, 92% of the plump kernels had tetraploid embryos whereas controls without treatment had 98% triploids (Kato 2001). In the treated material, 5.2% of the ovules produced plump kernels whereas only 1.5% did in the untreated control (Kato 2001). These experiments demonstrate that diploid sperm can function and the viable kernels resulting occur via heterofertilizations.

In the pollen from the High Loss line with B chromosomes, there is a mixture of pollen with a single diploid sperm and those with two haploid sperms. Heterofertilizations would give a normal endosperm development with fertilization by a haploid sperm with the other fertilization event receiving sperm of either ploidy from another pollen grain. The frequency of diploid sperm in the pollen is much higher than the frequency of triploids in the progeny, suggesting that heterofertilization is the limiting factor because its frequency is much lower (Gao et al. 2011).

The production of trisomies from the High Loss line with B chromosomes was present in our material. It is difficult to document the presence of two homologous chromosomes in examined pollen, but it is conceivable that these cases also result from the adherence of individual chromosomes at the second pollen mitosis. The presence of trisomies was not restricted to chromosomes containing cytologically detectable knobs, so the possibility exists that the adherence in these cases operates at the centromeric regions of the affected A chromosomes. This raises the possibility that highly repetitive sequences in general, in the case of centromeres, the CentC repeat (Ananiev et al. 1998b), are involved in this phenomenon. In this regard, the presence of high numbers of B chromosomes in the B73 background generates trisomies for various A chromosomes. We did not observe any triploids in this material or observe high rates of chromosomal deficiencies. Thus, the spill-over effect of the B chromosome in High Loss and B73 appears to be distinct. As noted above, neither chromosomal breakage nor trisomies are observed in W22 with B chromosomes even though this inbred line has several knobs (Albert et al. 2010).

Various authors have noted a negative correlation between high knob numbers and the presence of B chromosomes in Native American collections and other lines (Longley 1938; Bianchi et al. 1963). Whether this is a reflection of the conflict between B chromosomes and the A chromosomes or whether it is a reflection of population structure is not known. If it is due to conflict, then the effect of the B chromosomes that is so dramatic in the High Loss line might be more general across the species but perhaps to a lesser extent. The nondisjunction property of the B chromosome is part of its drive mechanism to be maintained but also results in a conflict with the host at least in some backgrounds. B73 has a reversal of preference fertilization favoring the B containing sperm fertilizing the central cell (Brennan et al. 2023), which would eliminate the B if its presence were not selected for. The High Loss line does not show this property. Whether the reversal of preference fertilization evolved as a reaction to the detrimental effects of the B chromosome is a question of interest but difficult to determine because various inbred lines were formed as conglomerates of distinct progenitor germplasm.

In this study we have documented the basis of the ploidy variation conditioned by the High Loss line with B chromosomes. The results indicate that the adherence of repetitive sequences at the second pollen mitosis in the High Loss background spills over from the B chromosome property of nondisjunction. Most lines of maize do not have this conflict being able to distinguish the B specific repeat for nondisjunction and the A chromosomal repetitive sequences that cause breakage or nondisjunction of A chromosomes. The eventual definition of the genes on the B chromosome that produce this effect and the gene or genes in the A chromosomes that differ from normal in the High Loss line will be of interest for further dissection of these phenomena.

## Supplementary information

Figure S1
