# Supplementary material for: Aneuploidy and ploidy variation conditioned by the B chromosome of maize
Source: Heredity (Edinb). 2025 Apr 25;135(8):563–70. doi: 10.1038/s41437-025-00764-y (PMC13545263; doi:10.1038/s41437-025-00764-y)
Supplement: Supplementary file 1 [file 41437_2025_764_MOESM1_ESM.pdf]

Supplementary Information, Figure S1.

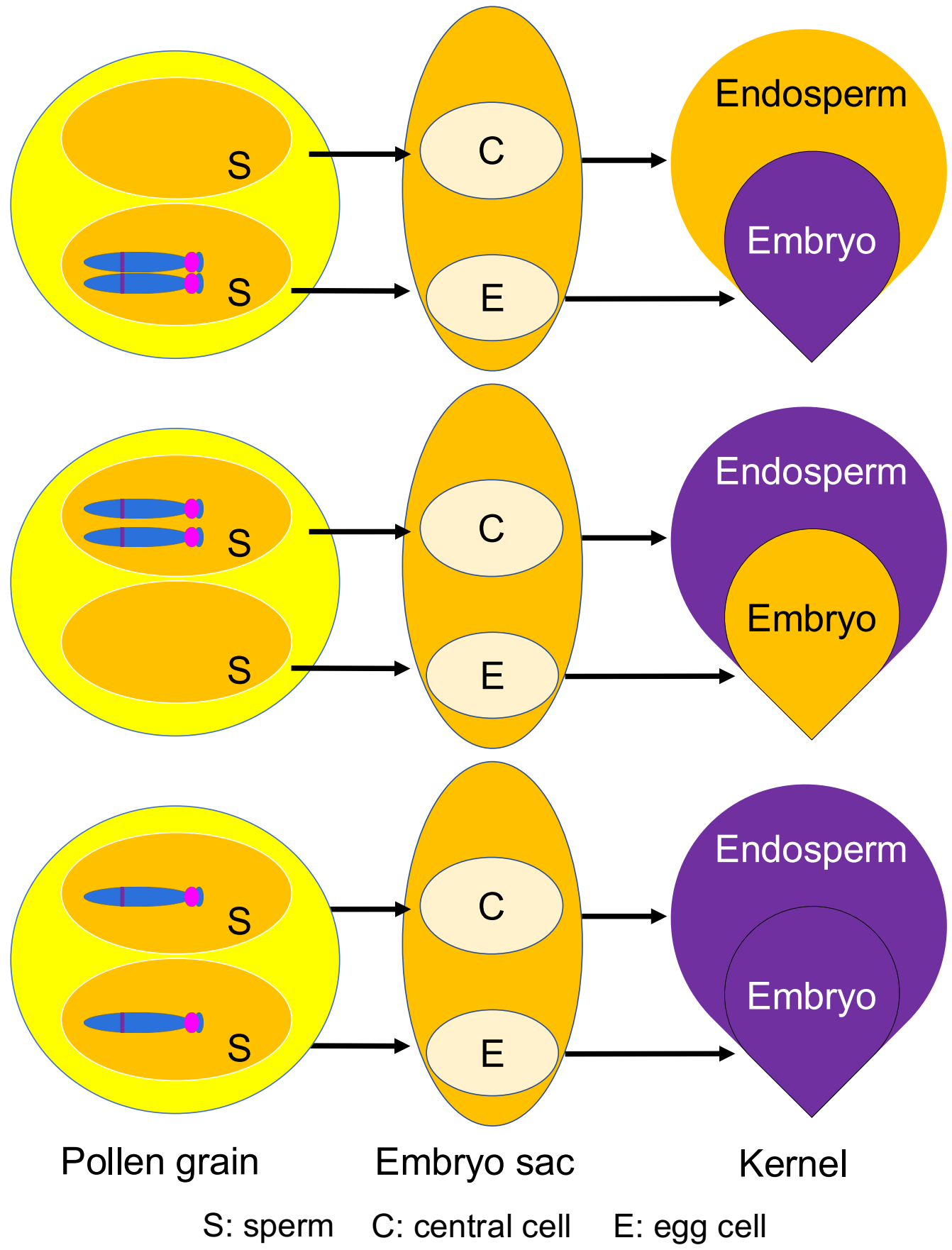

Fig S1. Diagram of nondisjunction and preferential fertilization (modified from Brennan et al 2023). B chromosomes containing an inserted B-Peru anthocyanin color marker (dark line) are shown in the sperm. Fertilization of the central cell gives rise to the endosperm, whereas fertilization of the egg cell gives rise to the embryo. Top panel: Depiction of preferential fertilization of the egg. The color marker is expressed in the embryo (purple). The endosperm is colorless (yellow). Middle panel: Preferential fertilization of the central cell, also known as reverse preferential fertilization, produces a colored endosperm and colorless embryo. Bottom panel: When nondisjunction of the B chromosomes does not occur, both the endosperm and embryo will express the color marker.

Brennan VA, Yang H, Gao Z, Birchler JA. (2023) Variation for maize B chromosome preferential fertilization: a component of the B chromosome drive mechanism. The Nucleus 66:305-310.
